# Supplementary figures and images for: Sex Determination Using RNA-Sequencing Analyses in Early Prenatal Pig Development
Source: Genes (Basel). 2019 Dec 5;10(12):1010. doi: 10.3390/genes10121010 (PMC6947224; doi:10.3390/genes10121010)

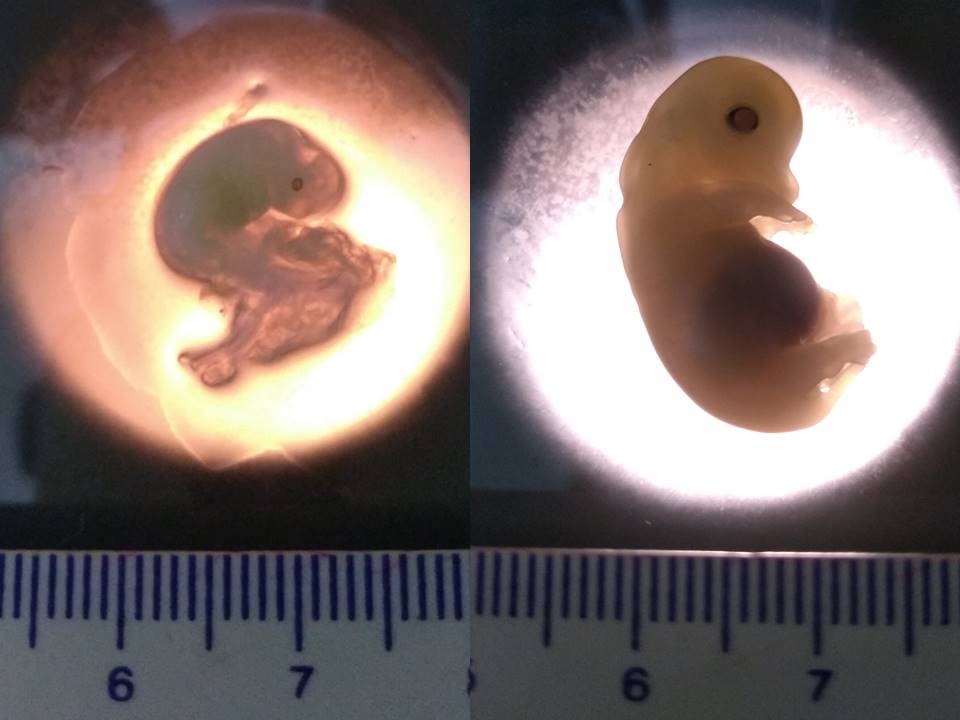

Supplement: Supplementary file 1 [file genes-10-01010-s001.zip › Figure S1.jpg]
